# Dopamine, Effort-Based Choice, and Behavioral Economics: Basic and Translational Research

**DOI:** 10.3389/fnbeh.2018.00052

**Published:** 2018-03-23

**Authors:** John D. Salamone, Merce Correa, Jen-Hau Yang, Renee Rotolo, Rose Presby

**Affiliations:** ^1^Department of Psychological Sciences, University of Connecticut, Storrs, CT, United States; ^2^Area de Psicobiologia, Universitat de Jaume I, Castelló, Spain

**Keywords:** motivation, reward, ventral striatum, accumbens, effort-related decision making, models, depression

## Abstract

Operant behavior is not only regulated by factors related to the quality or quantity of reinforcement, but also by the work requirements inherent in performing instrumental actions. Moreover, organisms often make effort-related decisions involving economic choices such as cost/benefit analyses. Effort-based decision making is studied using behavioral procedures that offer choices between high-effort options leading to relatively preferred reinforcers vs. low effort/low reward choices. Several neural systems, including the mesolimbic dopamine (DA) system and other brain circuits, are involved in regulating effort-related aspects of motivation. Considerable evidence indicates that mesolimbic DA transmission exerts a bi-directional control over exertion of effort on instrumental behavior tasks. Interference with DA transmission produces a low-effort bias in animals tested on effort-based choice tasks, while increasing DA transmission with drugs such as DA transport blockers tends to enhance selection of high-effort options. The results from these pharmacology studies are corroborated by the findings from recent articles using optogenetic, chemogenetic and physiological techniques. In addition to providing important information about the neural regulation of motivated behavior, effort-based choice tasks are useful for developing animal models of some of the motivational symptoms that are seen in people with various psychiatric and neurological disorders (e.g., depression, schizophrenia, Parkinson’s disease). Studies of effort-based decision making may ultimately contribute to the development of novel drug treatments for motivational dysfunction.

## Behavioral Economics, Choice and the Neural Regulation of Motivated Behavior

It is something of a truism that the field of economics is not about money, but rather, it is about choice. As it turns out, much of the study of motivation is also about understanding the neural basis of choice, which ultimately means that economic concepts and approaches can enlighten and inform research on the neurobiology of motivation (Salamone et al., 2016a,c, 2017). There is a rich literature on behavioral economics that has emerged from the field of experimental behavior analysis (e.g., Hursh and Winger, 1995; Bickel et al., 2000; Madden et al., 2000, 2007), which has provided useful terms and methods that can aid neuroscientists. For example, the results of some instrumental behavioral experiments, including those focusing on drug reinforcers, can be analyzed by quantative economic methods such as demand curves (Hursh and Winger, 1995; Heyman, 2000; Salamone et al., 2009; Madden and Kalman, 2010; Heinz et al., 2012; Bentzley et al., 2013, 2014; Bentzley and Aston-Jones, 2015).

According to the classical view of the reinforcement of instrumental behavior, a reinforcer is a stimulus that strengthens a response. Thus, a positive reinforcer is a stimulus that, when presented after a response, increases the probability or frequency of that response (Skinner, 1953). However, there are additional views of reinforcement that also are necessary to understand in order to explicate the deeper significance of this process. Positive reinforcers are typically motivational stimuli that the organism is likely to approach, or seeking to attain, and which would tend to occur with a relatively high probability in an unconstrained environment (Thorndike, 1911; Premack, 1959; Salamone and Correa, 2002). Furthermore, viewed from the perspective of economics, a reinforcer is a good or commodity, or an object or activity that has relatively high value. Given this background, what is an instrumental behavior such as pressing a lever, running in a maze, or pressing a computer keyboard? According to economic principles, an instrumental behavior is the labor that is exchanged for the good or commodity (i.e., the reinforcer). Thus, positively reinforced behavior is essentially a barter system, in which the organism trades its work for access to the reinforcer (Rachlin, 2003; Salamone et al., 2009). It therefore appears that instrumental response requirements are the price (i.e., the response cost) that needs to be paid in order to obtain access to motivationally relevant stimuli that are serving as reinforcers.

There is no shortage of articles in behavioral neuroscience that focus on “reward” or “reward value” or “outcome valuation”. The dopamine (DA) hypothesis of “reward” has been a ubiquitous feature of psychopharmacology and behavioral neuroscience for decades (e.g., but see also Salamone et al., 2007 and Nicola, 2016 for problems with this hypothesis). Decision making studies in animal subjects and human participants that investigate the impact of reinforcers differing in quality or quantity, or factors such as reinforcement delay or probability, have contributed greatly to our understanding of the neural regulation of motivated behavior (Floresco, 2015; Wassum and Izquierdo, 2015; Winstanley and Floresco, 2016). Nevertheless, it can be argued that the study of the other side of the equation, the labor or cost side, is equally important (Salamone and Correa, 2012; Wassum and Izquierdo, 2015; Salamone et al., 2016a,c; Winstanley and Floresco, 2016). To this end, several laboratories including our own have been focusing on the neural regulation of choice based upon exertion of physical effort. The work performed in order to obtain access to reinforcing stimuli is itself an important factor in regulating instrumental behavior, one which should not be subsumed or hidden under the general umbrella of “reward value”, but rather should receive its own spotlight. It is not as though the brain merely functions to assess the value of stimuli, and the actions necessary to obtain those stimuli are just some epiphenomena. It trivializes the neural regulation of instrumental behaviors to consider them merely as a small part of the “rewarding outcome”, when in fact they are the actions that lead to the outcome. Importantly, there is considerable evidence that manipulations affecting brain functions, such as drugs or lesions, can dissociate the exertion of effort in instrumental behavior from reinforcement value based upon preference (Salamone et al., 2016a,c, 2017). The present review will discuss some of this research, with a particular focus on the role of forebrain circuits and neurotransmitters such as DA, adenosine and GABA. Furthermore, this review will briefly describe how research on effort-based choice behavior can lead to the development of animal models that are useful for understanding aspects of psychopathology.

## Effort-Related Choice and Nucleus Accumbens DA

### Conceptual Background

The processes involved in initiating and sustaining instrumental actions, including the exertion of effort needed to overcome obstacles and obtain access to motivationally relevant stimuli, are necessary for survival. However, a complex environment can involve potential access to several different reinforcers, and distinct paths for accessing them. For these reasons, organisms must make choices involving several factors, including cost/benefit assessments based upon work requirements and reinforcement preference (Salamone and Correa, 2002, 2012; Walton et al., 2006; Salamone et al., 2007, 2016a,b,c; Winstanley and Floresco, 2016). Considerable evidence indicates that nucleus accumbens DA, along with other transmitters and structures, participates in the neural circuitry that regulates effort-based choice behavior (Salamone et al., 2007, 2009, 2016a,b,c; Floresco et al., 2008a; Hauber and Sommer, 2009; Mai et al., 2012; Floresco, 2015; Winstanley and Floresco, 2016). The effects of interfering with DA transmission have been assessed in many ways (Salamone et al., 2016a,b,c), including systemic or intracranial injections of DA antagonists, local depletions of accumbens DA with injections of the neurotoxic agent 6-hydroxydopamine (6-OHDA), or systemic administration of the DA depleting agent tetrabenazine, which blocks vesicular storage by inhibiting the type-2 vesicular monoamine transporter (VMAT-2). Moreover, various pharmacological, genetic, and optogenetic methods have been used to determine the effects of augmenting DA transmission.

### T-Maze Choice Procedures

One of the procedures that has been used to assess the contribution of accumbens DA to response allocation and effort-related choice behavior is a T-maze barrier choice procedure developed by Salamone et al. (1994). With this procedure, the two choice arms of the maze can have different reinforcement densities (e.g., 4 vs. 2 food pellets, or 4 vs. 0), and under some conditions a vertical barrier is placed in the arm with the higher density of food reinforcement to present an effort-related challenge. Under conditions in which the high-density arm (4 pellets) had the barrier in position, and the arm without the barrier contained an alternative food source (2 pellets), DA depletions or antagonism substantially alter effort-based choice, decreasing selection of the high-density arm, while increasing choice of the low-density arm with no barrier (Salamone et al., 1994; Cousins et al., 1996; Denk et al., 2005; Mott et al., 2009; Yohn et al., 2015a,b). The T-maze barrier choice task has undergone considerable behavioral validation and evaluation (Salamone et al., 1994; Cousins et al., 1996; van den Bos et al., 2006; Ostrander et al., 2011; Pardo et al., 2012; Yohn et al., 2015a,b). If there is no barrier obstructing the arm with the high reinforcement density, rats mostly choose that arm, and neither D1 or D2 family antagonists, nor accumbens DA depletions, nor tetrabenazine alter arm choice (Salamone et al., 1994; Yohn et al., 2015a,b). When the arm with the barrier contains 4 pellets, but the other arm contains no pellets, and thus the only way to obtain food is to climb the barrier, rats with DA depletions still choose the high-density arm, climb the barrier, and eat the pellets (Cousins et al., 1996; Yohn et al., 2015a). In a mouse study, although the DA antagonist haloperidol produced a low-effort bias when the high reward arm had a barrier, it had no effect on choice when both arms had a barrier in place (Pardo et al., 2012). Thus, interference with DA transmission did not alter preference for the high density of food reward over the low density, did not affect discrimination or reference memory processes related to arm preference, and did not produce an absolute impairment in the ability to climb the barrier.

### Lever Pressing Choice Procedures

Another commonly used task for assessing effort-based choice is the concurrent lever pressing/chow feeding procedure. With this task, rats are offered the option of either lever pressing to obtain a relatively preferred food (e.g., Bio-serv pellets; usually obtained on a fixed ratio 5 (FR5) schedule), or approaching and consuming a less preferred food (lab chow) that is concurrently available in the chamber. Well trained rats under baseline conditions typically get most of their food by lever pressing, and consume only small quantities of chow (Salamone et al., 1991, 2002). Low-to-moderate doses of DA D1 or D2 receptor antagonists produce a substantial shift in response allocation in rats performing on this task, decreasing lever pressing for food but substantially increasing intake of the concurrently available chow (Salamone et al., 1991, 2002; Cousins et al., 1994; Sink et al., 2008; Worden et al., 2009). This low-effort bias is also induced by local intra-accumbens injections of DA antagonists, neurotoxic depletions of accumbens DA, and tetrabenazine (Salamone et al., 1991; Cousins et al., 1993; Koch et al., 2000; Nowend et al., 2001; Farrar et al., 2010; Nunes et al., 2013a). As with the T-maze task, the use of the concurrent FR5/chow feeding choice task for assessing effort-related choice behavior has been validated in many ways. For example, the low doses of DA antagonists or tetrabenazine that produce the low-effort bias in effort-based choice did not affect total food intake or alter preference between these two specific foods in free-feeding choice tests (Salamone et al., 1991; Nunes et al., 2013a). The effects of DA antagonism or depletion were not mimicked by appetite suppressants belonging to several different classes, including amphetamine (Cousins et al., 1994), fenfluramine (Salamone et al., 2002) and cannabinoid CB1 antagonists (Sink et al., 2008), which failed to increase chow intake at doses that suppressed lever pressing, and in fact tended to decrease chow intake. In addition, reinforcer devaluation by pre-feeding to reduce food motivation suppressed both lever pressing and chow intake (Salamone et al., 1991). In more recent series of experiments, rats were given a choice between lever pressing on a FR7 schedule for a high concentration of sucrose vs. approaching and consuming a less preferred lower concentration (Pardo et al., 2015). In that study, tetrabenazine shifted choice behavior, decreasing lever pressing but substantially increasing intake of the lower concentration of sucrose that was concurrently available. These effects of tetrabenazine were seen at doses that did not alter preference between the two sucrose solutions, and did not blunt the appetitive taste reactivity (sometimes referred to as hedonic reactivity) induced by sucrose (Pardo et al., 2015). Taken together, these findings demonstrate that interference with DA transmission under conditions that suppress instrumental actions does not simply reduce appetite or primary food motivation. Rather, these manipulations alter the allocation of instrumental responses in a manner that interacts with the response requirement rather than the particular quality or quantity of the food reinforcer. Thus, rodents with compromised DA transmission still maintain fundamental aspects of food motivation, and are still directed towards the acquisition and consumption of food, but they have a low-effort bias and select an alternative, less effortful path to obtain food.

## Effort Discounting and Progressive Ratio/Chow Feeding Choice Procedures Offer Insights into the Effort-Related Motivational Functions of DA

### Effort Discounting Procedures

In addition to the behavioral tasks described above, several other procedures have been developed in order to assess effort-related motivational processes. Bardgett et al. (2009) developed an effort discounting task based upon the T-maze barrier procedures, and reported that D1 and D2 antagonism produced a low-effort bias. Floresco et al. (2008b) studied the effects of the DA antagonist flupenthixol using a discrete trial ratio discounting procedure in which rats could either emit a single press to receive two pellets (low-reward lever), or emit 2, 5, 10 or 20 responses to obtain four pellets (high effort/high-reward lever). Flupenthixol shifted effort discounting, reducing selection of the high effort lever in a manner that was independent of any effects of delay of reinforcement. In subsequent studies using this procedure it was shown that the effects of DA D1 or D2 receptor antagonism were characterized by actions on discounting based upon physical effort (ratio discounting) but not cognitive effort discounting (Hosking et al., 2015). To the extent that waiting for a reward involves some type of cognitive effort, it is interesting to note that local injections of DA antagonists into nucleus accumbens did not affect progressive interval responding (Wakabayashi et al., 2004). Robles and Johnson (2017) found that intraventricular injection of the D2 antagonist eticlopride altered effort-based decision making as assessed using a mouse two-lever choice task.

### Progressive Ratio Choice Procedures

Another procedure for assessing effort-based choice is a variant of the lever pressing/chow feeding task, which employs a progressive ratio (PROG) schedule as the lever pressing component. In order to understand this task, it is useful to provide some background on PROG operant schedules. With PROG schedules, the ratio requirement increases as successive ratios are completed, and responding continues until a “break point” occurs, at which point the animal essentially ceases to respond for a period of time. Determination of PROG break points can be a very useful tool for characterizing some of the actions of drugs that are self-administered, and for comparing self-administration behavior across different drugs or drug classes (e.g., Marinelli et al., 1998; Woolverton and Ranaldi, 2002). However, attaching conceptual or theoretical significance to the results of studies involving PROG schedules can be somewhat complicated. Although it is sometimes common to see PROG break points used as a measure of “reward”, it should be emphasized that break points do not actually provide a simple measure of the quality or quantity of a reinforcing stimulus in a manner that is uncontaminated by other factors (Salamone et al., 2009). In fact, drug or lesion-induced changes in PROG break points can reflect much more than effects on the appetitive motivational properties of a reinforcing stimulus (Arnold and Roberts, 1997; Hamill et al., 1999). For example, response-related factors such as changing the kinetic requirements of the instrumental response (e.g., increasing the height of the lever) was shown to decrease progressive ratio break points (Skjoldager et al., 1993; Schmelzeis and Mittleman, 1996). Rather than providing a direct or unambiguous measure of the appetitive motivational characteristics of a stimulus, PROG break points are more directly a measure of how much work the organism will do in order to obtain that stimulus (Stewart, 1974). Thus, it is reasonable to view PROG performance as resulting from effort-related decision-making processes, in which the organism is making a cost/benefit choice about whether or not to continue to respond. Such choices are based in part on factors related to characteristics of the reinforcer itself, and homeostatic factors, but also involve assessments of the work-related response costs and time constraints imposed by the ratio schedule (Salamone et al., 2009).

**Figure 1:**
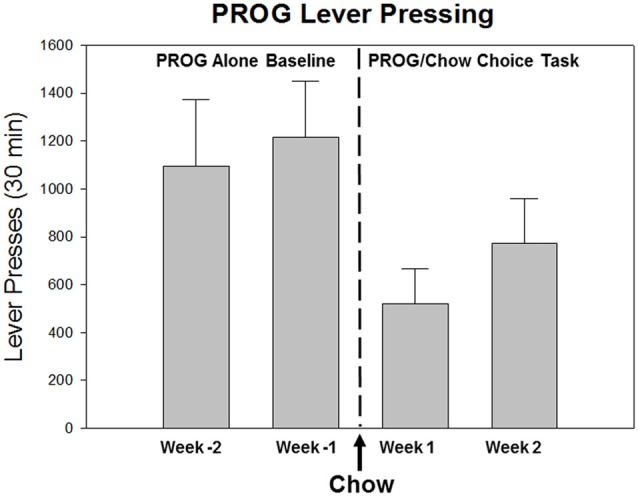
Effects of the availability of a food substitute on PROG lever pressing output reinforced by high-carbohydrate pellets. As described in the text, the PROG/chow feeding choice task is one of the behavioral procedures that is used to assess effort-based choice in rodents (Randall et al., 2012, 2014; Yohn et al., 2016c). This figure presents baseline training data from male Long Evans rats (*n* = 8) over the last 2 weeks of PROG alone training (Weeks -2 and -1) followed by the first 2 weeks of PROG/chow feeding choice training. As rats transition from the PROG alone schedule to the PROG/chow feeding choice task, in which an alternative food source (laboratory chow) is concurrently available in the chamber, it can be seen that the presence of the available chow significantly suppresses lever pressing output (*F*_(3,21)_ = 17.018, *p* < 0.001). The available chow is acting like a low-cost substitute that shifts demand away from the high-cost Bio-serv pellets that can only be obtained by working on the PROG schedule.

Consistent with these concepts, effort-based decision-making procedures have been developed that offer organisms a choice between lever pressing on a PROG schedule to obtain a preferred reinforcer vs. approaching and consuming the concurrently available but relatively less preferred chow (Schweimer and Hauber, 2005; Randall et al., 2012). Rats trained on this procedure begin each session by pressing the lever, but as the ratio requirement gradually increases, they eventually stop lever pressing and switch to chow. In behavioral economic terms, the chow is serving as a low-cost substitute that is obtained when the response costs of lever pressing are too high; thus, the presence of concurrently available chow suppresses the PROG lever pressing (Figure 1). Because of the progressively incrementing work requirement and the availability of a substitute, the baseline level of lever pressing is relatively low for most animals (though there is considerable variability; see below), and chow intake is relatively high, compared to performance on the FR5/chow feeding choice task. In the most commonly used version of this task a time out is employed (Randall et al., 2012, 2014, 2015), so that if the animal goes 2 min without receiving a reinforcement, the lever pressing option is no longer reinforced. The highest ratio achieved up to this time out thus serves as a measure that is equivalent to the break point. In a sense, the PROG/chow feeding choice procedure serves as a kind of ratio discounting procedure, in which the animal lever presses up to a point and then switches to the concurrently available chow when the lever pressing requirement is too high.

**Figure 2:**
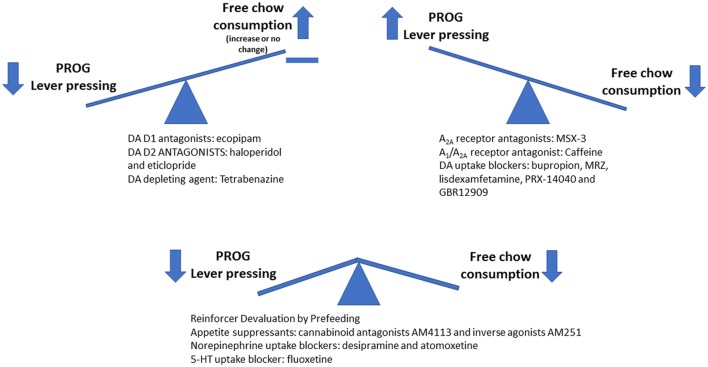
Schematic drawing summarizing the effects of various pharmacological manipulations on PROG/chow feeding choice performance. Interference with dopamine (DA) transmission by giving DA antagonists or tetrabenazine decreases PROG lever pressing but does not suppress chow intake. In fact, chow intake was significantly increased by the D1 antagonist ecopipam, and also increased in animals treated with haloperidol and tetrabenazine that had high baseline rates of lever pressing (Randall et al., 2012, 2014). In contrast, interfering with the unconditioned reinforcing properties of food by reinforcer devaluation (pre-feeding) or by administration of appetite suppressant drugs (CB1 receptor antagonists/inverse agonists) decreases both PROG lever pressing and chow intake. Finally, blockade of adenosine A_2A_ receptors or inhibition of DA uptake results in increased levels of PROG lever pressing (Randall et al., 2015; Yohn et al., 2016c).

In rats tested on the PROG/chow feeding choice task, lever pressing and highest ratio achieved (i.e., break point) are suppressed by administration of the DA D1 antagonist ecopipam and the D2 antagonists haloperidol and eticlopride (Randall et al., 2012, 2014). Although this result in itself may not be viewed as very surprising given what is known about DA, what is most important to consider is that despite these drug-induced decreases in lever pressing, intake of the concurrently available chow was not suppressed from its relatively high control levels, and in fact tended to increase still further (Randall et al., 2012, 2014). In contrast, the manipulations that fundamentally blunt the reinforcing characteristics of food, such as reinforcer devaluation by prefeeding, or administration of cannabinoid receptor antagonists or inverse agonists that are known to act as appetite suppressants (AM4113 and AM251), strongly suppress both lever pressing and chow intake (Randall et al., 2012, 2014). Lever pressing work output is also attenuated by the VMAT-2 inhibitor and DA depleting agent tetrabenazine (Randall et al., 2014), at doses that have no effect on food intake or preference between the two foods used in the PROG/chow feeding choice task (Nunes et al., 2013a). Thus, DA antagonism and depletion are not reducing PROG lever pressing because of a general suppression of the appetitvely motivating or unconditionally reinforcing characteristics of food (Figure 2). Instead, these manipulations effectively dissociate the tendency to work for food from the unconditioned reinforcing value of food as expressed by measures of intake and preference (Salamone et al., 2016a,b,c, 2017).

### Progressive Ratio Choice and Bidirectional Dopaminergic Control of Effort-Based Choice

Because of the relatively low baseline levels of lever pressing emitted by rats responding on the PROG/chow feeding choice task, this procedure provides an excellent baseline for assessing the effects of drugs that have the potential to increase selection of the high-effort PROG lever pressing (Figure 2). The adenosine A_2A_ receptor antagonist MSX-3, which has some of the characteristics of minor stimulants such as caffeine, was reported to increase PROG lever pressing and decrease chow intake (Randall et al., 2012). A high effort bias (i.e., towards PROG lever pressing) also has been seen after administration of the catecholamine uptake blocker bupropion (Randall et al., 2015), and the DA uptake blockers MRZ-9547 (Sommer et al., 2014), lisdexamfetamine (Yohn et al., 2016e), PRX-14040 (Yohn et al., 2016d) and GBR12909 (Yohn et al., 2016c). In contrast, the norepinephrine (NE) uptake blockers desipramine and atomoxetine and the 5-HT uptake blocker fluoxetine all failed to increase PROG lever pressing, in fact tending to suppress lever pressing (Yohn et al., 2016a).

These findings showing that pharmacological enhancement of DA transmission can increase selection of high-effort lever pressing illustrate the importance of DA transmission as a bi-directional regulator of effort-based choice, which is supported by research using non-pharmacological methods as well. Previous studies have observed that there was enhanced selection of high-effort instrumental actions in mice with knockdown of DA transporters (Cagniard et al., 2006), and also in mice that have increased expression of DA D2 receptors in nucleus accumbens that was induced in adulthood (Trifilieff et al., 2013). Recently, it was reported that selective chemogenetic activation of mesolimbic DA neurons, but not nigrostriatal neurons, increased responding for sucrose in rats responding on a PROG schedule (Boekhoudt et al., 2018), an effect that was characterized by increased initiation of bouts of instrumental behavior. Fischbach-Weiss et al. (2018) found that optogenetic inhibition of ventral tegmental area (VTA) DA neurons suppressed both the initiation and the maintenance of effortful operant responding on FR8 and PROG schedules. Furthermore, individual differences in performance of high-effort activities may be related to natural variations in DA transmission. As noted above, PROG/chow feeding choice performance is characterized by substantial individual differences that are very stable over time. Evidence indicates that rats with high PROG lever pressing output had significantly higher levels of phosphorylated DARPP-32 (DA and cAMP regulated phosphoprotein) in nucleus accumbens core neurons compared to low responders (Randall et al., 2012). This indicates that higher levels of DA-related signal transduction in post-synaptic medium spiny neurons are associated with a greater tendency to select high-effort instrumental activities. It is not clear if this finding represents a trait difference between high vs. low responders, or is instead a marker of having emitted a large number of responses. Nevertheless, an association between instrumental response output and pre- or postsynaptic markers of DA-related signaling, as seen in other studies (Sokolowski et al., 1998; Segovia et al., 2011, 2012; Howe et al., 2013; Saddoris et al., 2015; Hamid et al., 2016; Ko and Wanat, 2016; Wood et al., 2017), provides additional evidence of the importance of DA transmission in the regulation of response vigor and work output.

## The Use of Effort-Based Choice Tasks as Preclinical Tools for Animal Models of Psychopathology

### Impaired Behavioral Activation and Effort-Related Decision-Making in Psychopathology

As well as providing insights into the neurochemical regulation of fundamental aspects of motivation, studies of effort-based choice are being used for modeling motivational symptoms seen in some psychiatric and neurological disorders. This development has been influenced by several factors. First, it has been recognized for some time that various psychopathologies are associated with motivational dysfunctions that reflect a lack of behavioral activation or low exertion of effort. In addition to showing positive symptoms such as hallucinations and delusions, schizophrenic patients also display negative symptoms that include motivational dysfunctions (Gard et al., 2009). Depressed patients have symptoms that include psychomotor retardation, fatigue and anergia, which are very debilitating and are related to a number of disease outcomes (Gullion and Rush, 1998; Tylee et al., 1999). Fatigue is also commonly reported in stroke patients (De Doncker et al., 2018) and people with Parkinson’s disease (Chong et al., 2015). Generally speaking, these motivational dysfunctions are highly treatment resistant. For example, 5-HT uptake blockers that are commonly used to treat depression are relatively ineffective at restoring normal motivational function (Cooper et al., 2014; Fava et al., 2014; Rothschild et al., 2014). Against this backdrop, there has been a recent surge of research on humans that has specifically focused on effort-related decision making, both in people with various pathologies and healthy control participants. Wardle et al. (2011) reported that amphetamine increased selection of high-effort choices in healthy controls. Individual differences in the selection of the high-effort choice were positively associated with the degree of striatal DA release as measured by an imaging marker (Treadway et al., 2012b). Mueller et al. (2018) recently found that administration of α-methyltyrosine, which inhibits catecholamine synthesis, disrupted the ability of healthy control subjects to effectively integrate effort requirements and reward availability. Furthermore, human studies have shown that alterations in effort-based decision making are associated with depression (Treadway et al., 2012a; Yang et al., 2014, 2016; Culbreth et al., 2017), schizophrenia (Gold et al., 2013; Culbreth et al., 2017) and Parkinson’s disease (Chong et al., 2015).

### Animal Models of Effort-Related Motivational Dysfunction

Based upon the foundation provided by animal research, as well as the emerging clinical literature discussed above, formal animal models of motivational pathologies have been developed, which employ tasks assessing response vigor and effort-based decision making (Salamone et al., 2006, 2015, 2016a,b,c; Simpson et al., 2011, 2012; Markou et al., 2013; Bailey et al., 2016; Der-Avakian et al., 2016). Some of this research has involved an assessment of the effort-related effects in rodents of conditions associated with depression in general, or with specific motivational symptoms such as anergia and fatigue. Furthermore, potential drug treatments have been a target for research in these models. Because stress is such an important factor in psychopathology, some studies have tried to determine the effects of stress on effort-based choice. Restraint stress has been shown to induce a low-effort bias as measured with an effort discounting task in rats (Shafiei et al., 2012), and the effort-related effects of stress involve the actions of corticotropin-releasing hormone (Bryce and Floresco, 2016).

Another condition that has been used to induce a low-effort bias in animal models is the VMAT-2 inhibitor tetrabenazine. As described above, tetrabenazine is useful in research because it is a pharmacological tool for depleting DA, however, it also is used clinically to treat Huntington’s disease, and in this context has been shown to induce psychiatric side effects in humans such as depression and fatigue (Frank, 2009, 2010, 2014; Guay, 2010). While tetrabenazine has been used to produce deficits in classical animal models of depression such as the forced swim test (Tadano et al., 2000; Wang et al., 2010), recent studies have shown that tetrabenazine can induce a low-effort bias in rats tested on the FR5/chow feeding choice (Nunes et al., 2013b; Yohn et al., 2016b, d,e), PROG/chow feeding choice (Randall et al., 2014), and T-maze barrier choice tests (Yohn et al., 2015a,b). Control experiments conducted to validate the use of tetrabenazine have shown that the effort-related effects of tetrabenazine were not due to actions such as loss of appetite, changes in preference for chow vs. pellets, or preference across different concentrations of sucrose, discrimination of reinforcement magnitude, hedonic reactivity for sucrose, or reference memory (Nunes et al., 2013a; Randall et al., 2014; Pardo et al., 2015; Yohn et al., 2015a). Given this pattern of results, tetrabenazine clearly has considerable utility for inducing effort-related deficits in animals, which can serve as a baseline for identifying and characterizing potential drug targets for the treatment of effort-related motivational symptoms (Salamone et al., 2016a,b,c).

### Animal Models: Drug Development

Several monoamine uptake inhibitors are currently being used to treat depression. 5-HT uptake blockers (i.e., SSRIs) are the most commonly used antidepressants, and while they are effective at treating mood dysfunction and rumination, they have limited success in terms of restoring motivational function (Cooper et al., 2014; Fava et al., 2014; Rothschild et al., 2014). Recent experiments have been conducted to determine if monoamine uptake inhibitors with different patterns of selectivity for 5-HT, NE and DA uptake are able to reverse the effort-related effects of tetrabenazine. Two commonly prescribed SSRIs are fluoxetine and citalopram, and both of these drugs were unable to attenuate the effects of tetrabenazine on FR5/chow feeding choice performance (Yohn et al., 2016b,e). The NE transport inhibitor desipramine also was studied, and like the SSRIs, it was unable to reverse the effects of tetrabenazine (Yohn et al., 2016b). Despite these negative results with inhibitors of 5-HT and NE uptake, several DA uptake inhbitors have been shown to be effective at reversing the effects of tetrabenazine on effort-related decision making. Bupropion (Welbutrin) is widely used as an antidepressant, and clinical studies have reported that this catecholamine uptake inhibitor can be more effective than SSRIs at treating fatigue symptoms (Papakostas et al., 2006; Cooper et al., 2014). Recent animal studies have shown that bupropion can reverse the effort-related effects of tetrabenazine in rats tested on the T-maze barrier choice tasks (Yohn et al., 2015a), as well as the FR5/chow feeding choice (Nunes et al., 2013b; Yohn et al., 2016b), and PROG/chow feeding choice tasks (Randall et al., 2014). Several other drugs that are capable of inhibiting DA transport (GBR12909, PRX-14040, lisdexamfetamine (Vyvanse), methylphenidate, and modafinil), also have been reported to attenuate the effort-related effects of tetrabenazine (Salamone et al., 2016c; Yohn et al., 2016b, d,e). Consistent with these findings with animal models, human studies have shown that amphetamine and methylphenidate, and the atypical DA transport inhibitor modafinil, can have positive effects on motivational symptoms in depressed patients (Stotz et al., 1999; Lam et al., 2007; Ravindran et al., 2008).

As described above, the relatively low baseline levels of lever pressing in animals tested on the PROG/chow feeding choice task makes this procedure useful for assessing the potential to increase selection of the high-effort PROG lever pressing. This line of research provides important information about the ability of drugs to produce a high-effort bias (i.e., to enhance PROG output) in the absence of tetrabenazine. Sommer et al. (2014) reported that the DA uptake inhibitor MRZ-9547 increased lever pressing in animals tested on the PROG/chow feeding choice task. Furthermore, many of the drugs that reverse the effort-related effects of tetrabenazine have been shown to increase selection of lever pressing in rats tested on the PROG/chow feeding choice procedure, including the catecholamine uptake blocker bupropion (Randall et al., 2015), and the DA uptake blockers lisdexamfetamine (Yohn et al., 2016e), PRX-14040 (Yohn et al., 2016d) and GBR12909 (Yohn et al., 2016c). As described above, these results highlight the role of DA in modulating exertion of effort, but they also point to dopaminergic manipulations as potential treatments for aspects of motivational dysfunction (Salamone et al., 2016a). However, neither the 5-HT uptake blocker fluoxetine nor NE transport inhibitors desipramine and atomoxetine were able to increase PROG lever pressing (Yohn et al., 2016a). Consistent with these findings, atomoxetine also was reported to have no effect on physical effort discounting (Hosking et al., 2015). Thus, despite studies showing that locus ceruleus neuron activity increases during exertion of physical effort (Varazzani et al., 2015), it does not appear that augmenting NE transmission pharmacologically increases selection of high effort activities. It is possible that locus ceruleus NE neuron activity is a correlate of the peripheral sympathetic activation during physical activity, or that it is correlated with attentional or other cognitive processes that are activated in parallel with the exertion of physical effort (Guillery et al., 2017).

Taking these results together, it appears that studies of effort-based decision making may ultimately contribute to the development of novel drug treatments for motivational dysfunction. In this regard, it is important to recognize that animal models of effort-based choice are not strictly speaking global models of depression, but instead are focused on modeling a particular behavioral function, and its neural basis, which may be relevant for understanding specific psychopathological symptoms. Thus, this line of research is potentially relevant for investigating motivational dysfunctions seen across many different disorders (e.g., negative symptoms of schizophrenia; see Simpson et al., 2011, 2012; Markou et al., 2013; Yohn et al., 2017a). Such an approach is consistent with the NIH RDoC (research domain criteria) initiative (Cuthbert and Insel, 2013), which focuses on identifying the neural circuits that underlie specific psychiatric symptoms.

## Summary, General Conclusions and Broader Implications

As discussed above, considerable evidence indicates that mesolimbic DA exerts a bi-directional control over exertion of physical effort and effort-based choice. Interference with DA transmission by DA antagonism or depletion produces a low-effort bias in rodents tested on effort-based choice tasks, while increasing DA transmission with drugs such as DA transport blockers tends to enhance selection of high-effort options. The results from these pharmacology studies are consistent with findings from recent articles using optogenetic, chemogenetic and physiological techniques. Furthermore, although the present review has focused mainly on drugs or neurotoxic lesions affecting mesolimbic DA transmission, it is important to emphasize that other signaling molecules in addition to DA, and several other brain areas, also are involved in regulating behavioral activation, response vigor, exertion of effort, and effort-based choice. These additional parts of the circuitry include neuromodulators and neurotransmitters such as adenosine (Font et al., 2008; Mingote et al., 2008; Farrar et al., 2010; Nunes et al., 2010), acetylcholine (Nunes et al., 2013b), and glutamate (Hutchison et al., 2017), and brain areas including prefrontal/anterior cingulate cortex (Walton et al., 2002, 2003, 2006; Schweimer and Hauber, 2005; Hart et al., 2017), and basolateral amygdala (Floresco and Ghods-Sharifi, 2007; Hart and Izquierdo, 2017). The GABAergic ventral striatopallidal pathway is a key connection in this circuitry (Mingote et al., 2008). Injections of behaviorally effective doses of the DA antagonist flupenthixol into nucleus accumbens core increases extracellular GABA in lateral ventral pallidum (Salamone et al., 2010), and injections of the GABA-A agonist muscimol into lateral ventral pallidum produces a low-effort bias similar to the effects of DA antagonism or depletion (Farrar et al., 2008; Mingote et al., 2008). Furthermore, chemogenetic inactivation of ventral striatopallidal neurons was reported to increase responding on a PROG schedule in mice (Carvalho Poyraz et al., 2016). In addition to acknowledging the distributed brain network that regulates effort-based choice, it also is important to recognize the role played by peripheral inflammation as a factor involved in regulating effort-related aspects of motivation (Nunes et al., 2014; Yohn et al., 2016a, 2017b).

Studies focusing on behavioral activation, response vigor and effort-based choice also have had an impact on computational neuroscience and economic models. Niv et al. (2007) offered a model describing the relation between increased levels of extracellular DA and response vigor. Phillips et al. (2007) proposed that the role of DA in cost/benefit analyses can be described by simple utility functions, and suggested that release of DA opens a window of opportunistic drive, in which the threshold cost expenditure to obtain reinforcers is decreased. In recent years, there has been a proliferation of modeling approaches that are intended to help characterize the roles of effort exertion and reinforcement processing in motivated behavior (Klein-Flügge et al., 2016; Białaszek et al., 2017; Pessiglione et al., 2017; Vassena et al., 2017), and disentangle exertion of effort from opportunity costs (Zénon et al., 2016). An aspect of behavioral economics that is useful for understanding the role of DA in effort-related aspects of motivation is the concept of elasticity of demand (Salamone et al., 1997, 2016a,c, 2017). Price elasticity of demand for a commodity (e.g., a reinforcer is a commodity in the context of operant behavior experiments) refers to the effect of changes in price on demand for that commodity. Low elasticity refers to a situation in which the subject is relatively insensitive to price changes, while higher levels of elasticity refers to conditions in which the sensitivity to price is greater. As described above, one way of controlling work-related response costs or prices in experiments involving instrumental behavior is to vary the ratio requirement of the lever pressing task. Previous work has shown that neurotoxic depletions of accumbens DA make animals more sensitive to the ratio requirements on ratio schedules (Aberman and Salamone, 1999; Ishiwari et al., 2004), and this type of effect has consistently been interpreted as representing a role for DA in mediating elasticity of demand for food (Salamone et al., 1997, 2009; Aberman and Salamone, 1999). Although these earlier studies did not provide mathematical indices of elasticity of demand, recent studies demonstrated that low doses of the DA antagonist haloperidol and the DA depleting agent tetrabenazine increased point elasticity of demand (Salamone et al., 2017). Taken together, these findings are consistent with a recent article reporting that DA D2 receptor knockout also increased elasticity of demand (Soto et al., 2016).

In addition to providing important basic science information about the neural regulation of motivated behavior, effort-based decision making research has several practical applications. Within the fields of industrial/organizational psychology and behavioral health, it is becoming more and more emphasized that effort/reward trade-offs can be a substantial source of stress in the workplace (Eddy et al., 2017, 2018). As reviewed above, studies of effort-related decision making in humans have the potential to offer important insights into motivational dysfunction in psychopathology. Furthermore, tasks that assess effort-based choice are useful for developing animal models of some of the motivational symptoms that are seen in people with various psychiatric and neurological disorders (e.g., depression, schizophrenia and Parkinson’s disease). Exertion of effort in pursuit of rewards is also a feature of drug self-adminitration in humans and other animals (Vezina et al., 2002; Venugopalan et al., 2011). Addicts will exert considerable effort to obtain their preferred drug, and will overcome numerous obstacles and constraints to do so. Furthermore, drug abstinence has been shown to be associated with psychomotor retardation and reduced selection of high-effort options in humans (Volkow et al., 2001) and animal models (Thompson et al., 2017).

Although it is often the case that animal models are designed to target neuropsychiatric disorders, it is nevertheless true that most animal models provide behavioral phenotypes that mimic specific symptoms or dysfunctions rather than entire disorders. Studies of effort-related choice, in so far as they contribute to animal models in psychiatry, are indeed designed to focus on modeling specific symptoms and circuit dysfunctions, and are not intended to provide global models of depression or schizophrenia. Nevertheless, this line of work may offer further insights into the neural circuits underlying motivational dysfunction in humans, and may also lead to the identification of drug targets that improve treatment outcomes for specific motivational symptoms that can be highly problematic and debilitating.

## Ethics Statement

This research was carried out in accordance with US NIH guidelines, and the protocol was approved by the University of Connecticut Institutional Animal Care and Use Committee.

## Author Contributions

JDS, MC, J-HY, RR and RP contributed to the writing of this manuscript. RR and MC also constructed the figures.

## Conflict of Interest Statement

The authors declare that the research was conducted in the absence of any commercial or financial relationships that could be construed as a potential conflict of interest.
